# The prognostic value of inflammation markers in postoperative gliomas with or without adjuvant treatments

**DOI:** 10.1097/MD.0000000000026437

**Published:** 2021-06-25

**Authors:** Yuanfu Luo, Renzhi Deng, Qiulu Zhong, Danjing Luo, Xiangde Li, Xueyuan Chen, Sha Tao, Zhoubin Feng, Liu Jiayi, Yiyun Huang, Jian Li, Wenqi Liu

**Affiliations:** a Department of Radiation Oncology; b Department of Neurosurgery, The Second Affiliated Hospital of Guangxi Medical University; c Guangxi Medical University, Nanning 530021, China.

**Keywords:** adjuvant treatments, gilomas, inflammation markers, NLR, PLR, SII

## Abstract

Recent studies have shown that some inflammatory markers are associated with the prognosis of solid tumors. This study aims to evaluate the prognosis of glioma patients with or without adjuvant treatment using the systemic immune-inflammation index (SII), neutrophil-to-lymphocyte ratio (NLR), and platelet-lymphocyte ratio (PLR).

All patients who were diagnosed with gliomas at the first and second affiliated hospital of Guangxi Medical University between 2011 and 2020 were included in this study. The optimal cutoff value of SII, NLR, and PLR was determined by X-tile software program. We stratified patients into several groups and evaluated the progression-free survival (PFS) and overall survival (OS) of SII, NLR, and PLR during the period of pre-surgical, con-chemoradiotherapy, and post-treatments. Multivariate Cox regression analyses were performed to detect the relationships between OS, PFS, and prognostic variables.

A total of 67 gliomas patients were enrolled in the study. The cutoff values of SII, NLR, and PLR were 781.5 × 10^9^/L, 2.9 × 10^9^/L, and 123.2 × 10^9^/L, respectively. Patients who are pre-SII < 781.5 × 10^9^/L had better PFS (*P* = .027), but no difference in OS. In addition, patients who had low pre-NLR (<2.9 × 10^9^/L) meant better OS and PFS. PLR after adjuvant treatments (post-PLR) was significantly higher than pre-PLR (*P* = .035). Multivariate analyses revealed that pre-SII, pre-NLR were independent prognostic factors for OS (pre-SII: HR 1.002, 95% CI: 1.000–1.005, *P* = .030 and pre-PLR: HR 0.983, 95% CI: 0.973–0.994, *P* = .001), while pre-PLR was an independent factor for PFS (HR 0.989, 95% CI: 0.979–1.000, *P* = .041).

High pre-SII or high pre-NLR could be prognostic markers to identify glioma patients who had a poor prognosis.

## 1 Introduction

Glioma is one of the most common primary intracranial tumors. According to the 2016 revision of the World Health Organization (WHO) classification of central nervous system tumors, gliomas can be classified into 4 grades. Grades I and II belong to low-grade gliomas, while grades IIII and IV are high-grade gliomas.^[1]^ Despite the safety resection followed by adjuvant chemoradiotherapy or radiotherapy of high-grade gliomas has always been the standard treatment, the prognosis is still poor and some cases recur within a short time. In Yang et al study, patients with grade III glioma or glioblastoma had a poor prognosis, the median overall survival (OS) for patients with anaplastic glioma was 37.6 months, whereas only 14.4 months for glioblastoma patient.^[2]^

Several studies have shown that chronic inflammation may be a major characteristic of the tumor microenvironment and may accelerate tumor progression or metastasis.^[3,4]^ Bambury et al found that neutrophil-lymphocyte ratio (NLR) is a marker of systemic inflammatory response and a poor prognostic factor in many malignancies such as colon, bladder cancer, and prostate cancer. The authors pointed out NLR > 4 was an independent factor for a poor prognosis of glioblastoma.^[5]^ Wang et al quantified the prognostic value of platelet-lymphocyte ratio (PLR), NLR, and lymphocyte-to-monocyte ratio (LMR) based on the IDH mutation status and pointed out low NLR was a better prognosis in IDH-wt glioblastoma group, while PLR was predictive of survival in patients with glioblastoma, pGBM, and IDH-wt GBM groups.^[6]^ Besides, the preoperative systemic immune-inflammation index (SII) is also being confirmed as a biomarker for predicting the survival and quality of life in patients with esophageal squamous cell carcinoma, small cell lung cancer, and gastric cancer.^[7–10]^ Li et al^[11]^ also compared the inflammatory and nutritional markers between preoperative neoadjuvant chemotherapy (NACT) and after NACT. The author found that NACT could decrease some inflammatory markers, whereas initial NLR, anemia, and LMR were poor prognosis in locally advanced gastric cancer. As we know, the prognosis of different grades of gliomas are varying. At present, the diagnosis of glioma is based on molecular types, which are of certain significance for the prognosis of glioma patients. However, it is almost impossible to re-biopsies to obtain molecular pathology during the treatment. If some tumor biomarkers which are related to prognosis can be acquired in peripheral blood, it will play an important role in selecting adjuvant therapy and evaluating the prognosis of glioma patients. SII, NLR, and PLR are based on the ratio of neutrophil, lymphocyte, and platelet counts in peripheral blood, and it is easy to get. So if these biomarkers have strong prognostic power for glioma patients, it could bring great benefits for patients with glioma. In previous studies, inflammatory markers such as SII, NLR, and PLR have been proven to be related to the prognosis of gliomas, but the relevant data are few, and more studies are needed to confirm the value of SII, NLR, and PLR as prognostic factors in glioma. Therefore, we aimed to explore the prognostic value of SII, NLR, and PLR at baseline and during the treatments. We defined patients into several groups to investigate the prognosis in gliomas.

## 2 Methods

### 2.1 Patient selection

We retrospectively collected all the patients who were diagnosed with gliomas between 2011 and 2020 at the first and second affiliated hospital of Guangxi Medical University. The inclusion criteria for patients were as follows: 1) all the glioma patients were confirmed by pathological examination, including oligodendroglioma, astrocytoma, anaplastic astrocytomas, olidendromas, and glioblastma; 2) patients ranged in age from 16 to 75 years old; 3) all the patients were initially treated without previous chemotherapy or radiotherapy; and 4) Karnofsky performance status (KPS) ≥70 scores. The exclusion criteria were as follows: 1) patients who had recently pyrexia (axillary ≥73.2°C); 2) any other form of active infection that may affect SII; 3) patients with chronic inflammatory disease; 4) patients who died from treatment-related complications;5) a history of tumor radiotherapy or chemotherapy;6) with second primary malignant disease; and 7) incomplete follow-up data. The clinical characteristics of all enrolled patients were shown in Table 1. The patient flowchart was clearly shown in Figure 7.

**Table 1:** Clinical characteristics of patients.

Clinical data	Value
Age (years)	
>40	37
≤40	30
Gender	
Male	38
Female	29
Tumor site	
Parietal lobe	8
Temporal lobe	15
Frontal lobe	38
Occipital lobe	2
Not otherwise specified	4
WHO stage	
I	4
II	24
III	16
IV	23
Pathology	
Low-grade glioma	28
High-grade glioma	39
Cycles for adjuvant chemotherapy	
<6	19
≥6	9
Adjuvant treatments	
Radiotherapy	9
Chemoradiotherapy	23
Chemotherapy	3
No	32
Tumor size (cm)	
<6	26
≥6	41

WHO = World Health Organization.

### 2.2 Data analysis

Clinical data were collected from medical records, including name, age, gender, preoperative-neutrophil, preoperative-lymphocyte, con-lymphocyte, con-platelet, post-neutrophil, post-lymphocyte, post-platelet, tumor size, tumor site, histopathological tumor grade, dose of gross tumor volume of tumor bed, dose of Clinical Tumor volume 1, dose of Clinical Tumor volume 2, WHO grade, adjuvant treatment methods (chemo-radiotherapy, radiotherapy, chemotherapy, or only surgical resection), and cycles of adjuvant chemotherapy. The endpoints included the OS, progression-free survival (PFS). The calculated methods were as follows: SII = platelets × neutrophils/lymphocytes, NLR = neutrophils/lymphocytes, and PLR = platelets/lymphocytes.

### 2.3 Statistical analysis

All the data were analyzed by SPSS version 22.0 software (IBM), X-tile software program (Version 3.6.1; Yale University, School of Medicine), and graphpad prism 5. Continuous variables were compared by Student *t* test when they were normally distributed, while non-normally distributed we used Wilcoxon rank-sum to test. We used X-tile software program (Version 3.6.1; Yale University, School of Medicine) as described previously^[12]^ to decide the cutoff value of pre-SII, NLR, and PLR. Differences of SII, NLR, PLR between pre-treatments, post-treatments, and recurrent-treatments (rec-treatments) were analyzed by paired *T* tests. Survival curves were drawn by graphpad prism 5. According to the cutoff value, patients were divided into the following groups: pre-SII ≥ 781.5 group, pre-SII < 781.5 group; pre-NLR ≥ 2.9 group and pre-NLR < 2.9 group; and pre-PLR≥123.2 group and pre-PLR < 123.2 group. The characteristic of each group was shown in Table 2. The Kaplan–Meier method was used to estimate the median value of PFS and OS. The endpoints were OS and PFS. Survival was defined as the time between diagnosis and death or the time of the last follow-up. The PFS time was defined as the time between diagnosis and the patient's recurrence or progression. Cox proportional hazards models were used to identify predictors of other covariates, such as treatment methods, disease stage, doses of radiation, etc. Two-tailed *P* value <.05 was considered a statistically significant difference.

**Table 2:** The relationship between variable SII, NLR, PLR, and clinical characteristics.

	Pre-SII			Pre-NLR			Pre-PLR		
Variables	≥781.5 (n = 21)	<781.5 (n = 46)	*P*	≥2.9 (n = 20)	<2.9 (n = 47)	*P*	≥123.2 (n = 38)	<123.2 (n = 29)	*P*
Age (years)									
>40	13	24	.457	12	25	.608	22	15	.615
≤40	8	22		8	22		16	14	
Gender									
Male	8	30	.038	8	30	.072	15	23	.001
Female	13	16		12	17		23	6	
Tumor site									
Parietal lobe	6	7	.076	2	7	.801	2	6	.167
Temporal lobe	5	12		5	10		7	8	
Frontal lobe	6	25		10	27		24	13	
OccipitallLobe	2	0		1	1		2	0	
Not otherwise specified	2	2		2	2		2	2	
WHO stage									
I	1	3	.410	1	3	.410	2	2	.599
II	7	17		7	17		14	10	
III	3	13		3	13		7	9	
IV	10	13		9	14		15	8	
Pathology									
Low-grade glioma	8	20	.679	8	21	.723	17	12	.783
High-grade glioma	13	26		12	26		21	17	
Cycles for adjuvant chemotherapy									
<6 weeks	6	13	.989	2	10	.028	11	8	.349
≥6 weeks	3	6		9	7		7	2	
No	12	27		9	30		20	19	
Adjuvant treatments									
Radiotherapy	0	9	.144	0	9	.191	5	4	.988
Chemoradiotherapy	7	16		7	16		13	10	
Chemotherapy	1	2		1	2		2	1	
No	13	19		12	20		18	14	
Tumor size (cm)									
<6	14	29	.774	15	28	.228	24	19	.842
≥6	7	17		5	19		14	10	

NLR = neutrophil-to-lymphocyte ratio, PLR = platelet-lymphocyte ratio, SII = immune-inflammation index, WHO = World Health Organization.

## 3 Results

### 3.1 Basic characteristics

A total of 67 glioma patients (38 males and 29 females) were enrolled in the study. The median followed-up time was 14.7 months (1.0–96.6 months). The median age of the patients was 42 years (16–17years). Within this study, 28 patients (41.8%) had low-grade gliomas and 39 patients (58.2%) had high-grade gliomas. In the research, there were 9 patients (13.4%) who received postoperative radiotherapy, 23 patients (34.3%) received chemoradiotherapy, only 3 cases (4.5%) of chemotherapy, and 32 cases (47.8%) of surgery without adjuvant therapy. By the time of the last follow-up, 32 patients were still alive (17 cases of low-grade gliomas and 15 cases of high-grade gliomas, respectively) and 35 patients had died (11 with low-grade gliomas and 24 with high-grade gliomas, respectively). As determined by X-tile software program, the cutoff values of pre-SII, NLR, and PLR were 781.5 × 10^9^/L, 2.9 × 10^9^/L, and 123.2 × 10^9^/L, respectively.

### 3.2 Features of pre-SII, pre-NLR, and pre-PLR

According to the cutoff values, we defined SII, NLR, and PLR into several groups. There were 21 patients’ pre-SII ≥ 781.5 × 10^9^/L and 46 patients <781.5 × 10^9^/L; 20 patients’ pre-NLR ≥ 2.9 × 10^9^/L and 47 patients <2.9 × 10^9^/L. For pre-PLR, there were 38 patients’ pre-PLR ≥ 123.2 × 10^9^/L, while 29 patients <123.2 × 10^9^/L. Most of the pre-SII < 781.5 × 10^9^/L were male, while the female had a similar distribution in the 2 groups (male: 8 vs 30; female:13 vs 16, *P* = .038). In the pre-PLR groups, the majority of females’ PLR before treatments were ≥123.2 × 10^9^/L, whereas males were <123.2 × 10^9^/L (female: 23 vs 6; male 15 vs 23, *P* = .001).

### 3.3 Changes of SII, NLR, and PLR before, after, and recurrent treatments

In this research, PLR was significantly increased after adjuvant treatments (*P* = .035), while SII and NLR were not significantly decreased (*P* = .646, *P* = .118, Table 3). There were no significant statistical differences in SII, NLR, and PLR before and after treatments for patients with recurrence (*P* = .654, 0.296, 0.198, respectively, Table 4).

**Table 3:** The comparisons of SII, NLR, and PLR between pre-treatments and post-treatments.

Markers	Pre-treatments	Post-treatments	*P* value
SII	893.77 (61.05–6110.88)	639.73 (71.64–2361.86)	.646
NLR	3.04 (0.66–20.58)	2.94 (0.39–11.14)	.118
PLR	160.41 (36.34–480.00)	190.19 (60.46–595.97)	.035

NLR = neutrophil-to-lymphocyte ratio, PLR = platelet-lymphocyte ratio, SII = immune-inflammation index.

**Table 4:** The comparisons of SII, NLR, and PLR between pre-treatments and recurrent-treatments.

Markers	Pre-treatments	Rec-treatments	*P* value
SII	893.77 (61.05–6110.88)	1420.97 (223.63–7941.93)	.654
NLR	3.04 (0.66–20.58)	4.80 (1.44–19.56)	.296
PLR	160.41 (36.34–480.00)	224.02 (67.97–712.28)	.198

NLR = neutrophil-to-lymphocyte ratio, PLR = platelet-lymphocyte ratio, SII = immune-inflammation index.

### 3.4 Prognostic value of pre-SII, pre-NLR, and pre-PLR

**Figure 1:**
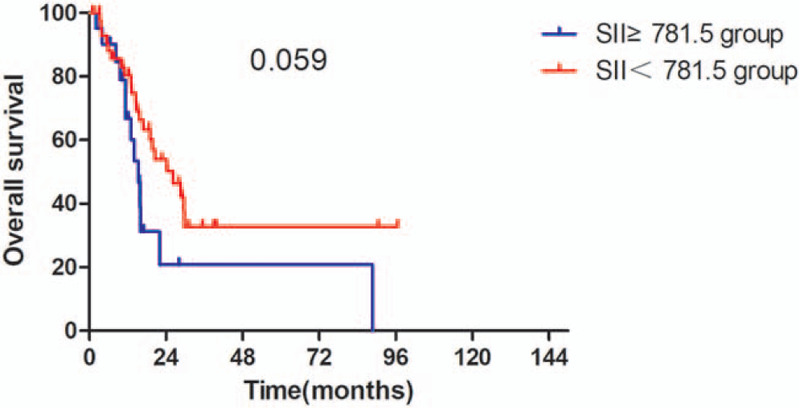
Kaplan–Meier curve for OS in glioma patients stratified by pre-SII. OS = overall survival, SII = immune-inflammation index.

**Figure 2:**
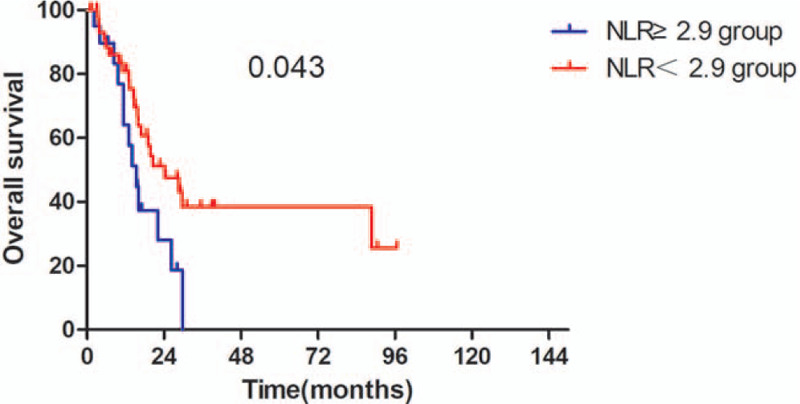
Kaplan–Meier curve for PFS in glioma patients stratified by pre-SII. PFS = progression-free survival, SII = immune-inflammation index.

**Figure 3:**
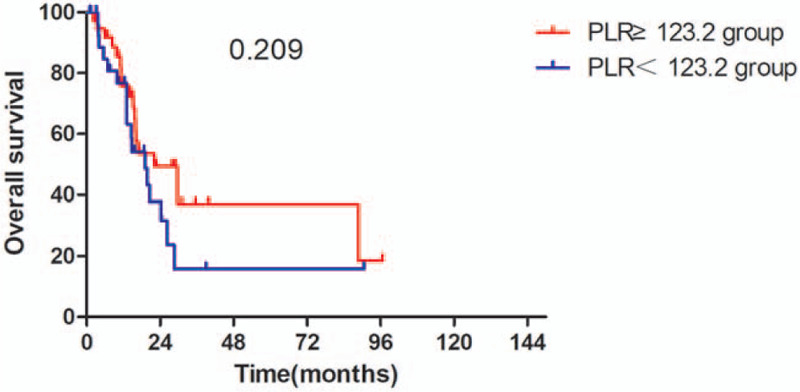
Kaplan–Meier curve for OS in glioma patients stratified by pre-NLR. OS = overall survival, NLR = neutrophil-to-lymphocyte ratio.

**Figure 4:**
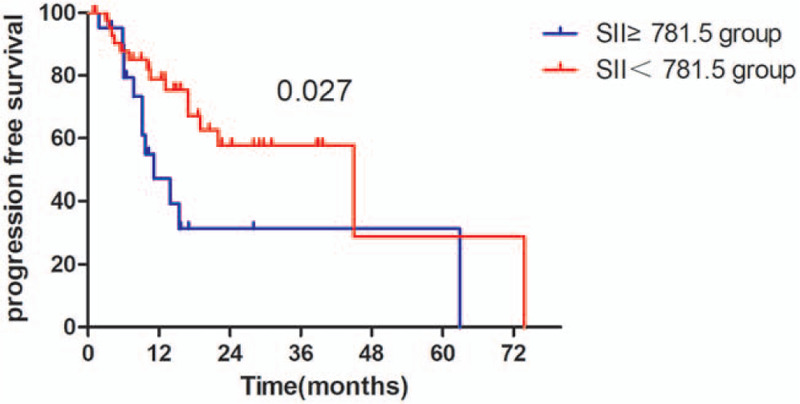
Kaplan–Meier curve for PFS in glioma patients stratified by pre-NLR. PFS = progression-free survival, NLR = neutrophil-to-lymphocyte ratio.

**Figure 5:**
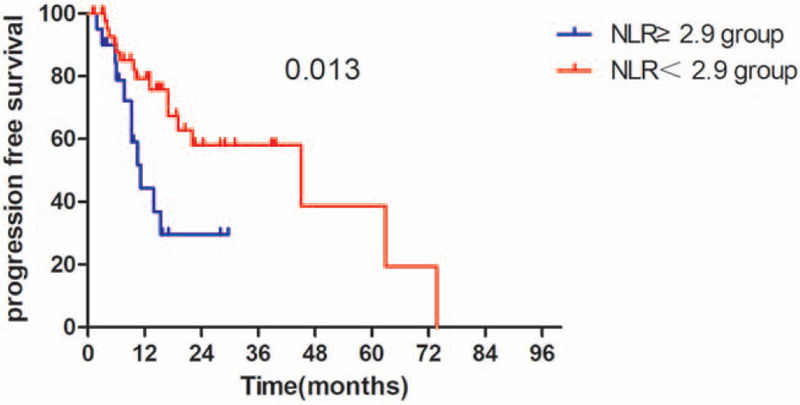
Kaplan–Meier curve for OS in glioma patients stratified by pre-PLR. OS = overall survival, PLR = platelet-lymphocyte ratio.

**Figure 6:**
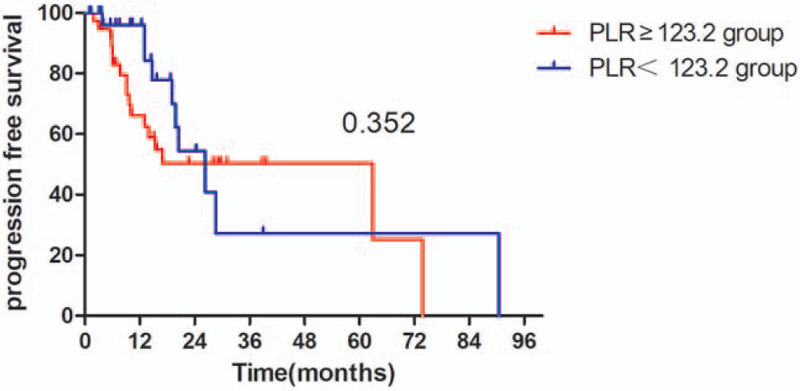
Kaplan–Meier curve for PFS in glioma patients stratified by pre-PLR. PFS = progression-free survival, PLR = platelet-lymphocyte ratio.

The Kaplan–Meier survival analyses demonstrated that the OS rate was similar in pre-SII ≥ 781.5 × 10^9^/L group and pre-SII < 781.5 × 10^9^/L group (*P* = .059, Fig. 1). The median OS was 15.3 months (95% CI 11.95–18.65 months) for patients with pre-SII ≥ 781.5 × 10^9^/L and 26.3 months (95% CI 16.14–36.46 months) for those with pre-SII < 781.5 × 10^9^/L. And the PFS of patients with pre-SII < 781.5 × 10^9^/L was significantly better than patients with pre-SII ≥ 781.5 × 10^9^/L (*P* = .027, Fig. 4). In addition, the OS and PFS of patients with pre-NLR < 2.9 × 10^9^/L were also significantly better than those with pre-NLR ≥ 2.9 × 10^9^/L (OS: *P* = .043; Fig. 2, PFS: *P* = .013, Fig. 5). As for PLR, there were no significant differences in OS and PFS of patients with pre-PLR ≥ 123.2 × 10^9^/L and those with pre-PLR < 123.2 × 10^9^/L (OS, *P* = .209; PFS, *P* = .352, Figs. 3 and 6).

**Figure 7:**
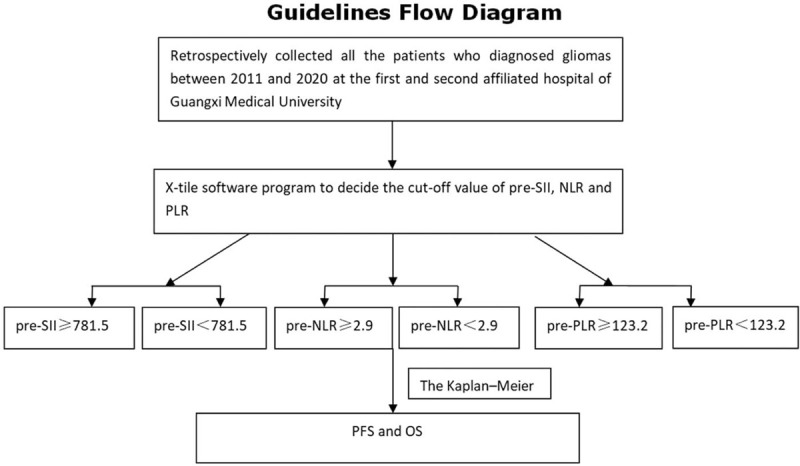
The patients flow chart.

### 3.5 Multivariate analyses

The Cox proportional hazards model analyses indicated that pre-SII, pre-PLR were significant independent factors of OS (pre-SII: HR 1.002, 95% CI: 1.000–1.005, *P* = .030; pre-PLR: HR 0.983, 95% CI: 0.973–0.994, *P* = .001) whereas the pre-PLR was a significant independent factor of PFS (HR 0.989, 95% CI: 0.979–1.000, *P* = .041, Tables 5 and 6).

**Table 5:** Multivariate analysis on overall survival.

Prognostic factors	Hazard ratio	95% CI	*P* value
Pre-SII	1.002	1.000–1.005	.030
Pre-NLR	0.922	0.443–1.922	.829
Pre-PLR	0.983	0.973–0.994	.001
Con-SII	0.999	0.995–1.003	.772
Con-NLR	1.463	0.594–3.603	.407
Con-PLR	1.003	0.988–1.018	.739
Post-SII	1.000	0.995–1.004	.930
Post-NLR	0.445	0.123–1.610	.217
Post-PLR	1.006	0.999–1.014	.107

CI = confidence interval, NLR = neutrophil-to-lymphocyte ratio, PLR = platelet-lymphocyte ratio, SII = immune-inflammation index.

**Table 6:** Multivariate analysis on progression-free survival.

Prognostic factors	Hazard ratio	95% CI	*P* value
Pre-SII	1.001	0.998–1.003	.626
Pre-NLR	1.437	0.641–3.224	.379
Pre-PLR	0.989	0.979–1.000	.041
Con-SII	1.001	0.998–1.004	.580
Con-NLR	1.328	0.568–3.105	.512
Con-PLR	1.006	0.992–1.020	.439
Post-SII	0.999	0.996–1.003	.641
Post-NLR	1.050	0.487–2.263	.900
Post-PLR	1.000	0.992–1.007	.981

CI = confidence interval, NLR = neutrophil-to-lymphocyte ratio, PLR = platelet-lymphocyte ratio, SII = immune-inflammation index.

## 4 Discussion

With the rapid development of molecular biology, the diagnosis and treatments of gliomas mainly depend on molecular biomarkers. However, due to the difficulty in obtaining tumor specimens during treatment, so we cannot monitor the molecular markers all the time. Most inflammatory markers are readily available, and it is convenient to detect the related biomarkers during the treatment period. Studies have shown that there is a certain relationship between inflammation and cancer, and immune cells play a predominant role in the of process inflammation, promoting tumor growth, angiogenesis, and metastasis.^[33]^ In recent studies, there have been several studies of SII, NLR, and PLR that have confirmed that these inflammations are associated with solid malignancies, such as esophageal carcinoma, gastrointestinal tumors, prostate cancer, and lung cancer.^[13–18]^ However, data on the prognostic value of SII, PLR, and NLR in gliomas is still limited, and most studies have mainly used inflammatory markers to distinguish low-grade gliomas from high-grade gliomas. Therefore, we aimed to know about the preoperative SII, NLR, and PLR levels of gliomas, and to evaluate the prognosis of gliomas by these markers. In our study, we found low preoperative SII was a better prognostic factor for PFS in patients with glioma. The results were similar to Liang et al study.^[19]^ As the hematological tumor markers were based on platelet, neutrophil, and lymphocyte counts, the prognostic value may be related to the varied functions of these cells. It has been shown that platelet-derived growth factor is a crucial role in glial tumorigenesis.^[34]^ And neutrophils are involved in the progress of promoting adhesion growth factors, seeding tumor through the secretion of circulating growth factors.^[35,36]^ In the study of Huang et al,^[20]^ high preoperative SII was associated with poor clinicopathological characteristics and poor prognosis of gastric cancer. Lolli et al^[21,22]^ evaluated the dynamic change of SII in renal cancer and prostate cancer. In this research, the authors divided patients into several subgroups based on the SII and its changes during the treatment. They found that SII might be a potential prognostic indicator. A single center in Kazakhstan studied 173 gliomas and they found that patients with glioblastoma (grade IV) had higher NLR than patients with grade I to III gliomas.^[23]^ Another study from Poland came to similar conclusions.^[24]^ As for PLR, the prognostic value of PLR still controversial in previous studies. Some studies have reported that increasing PLR may decrease OS in solid tumors such as lung cancer and gastric cancer.^[25–27]^ In our research, we found the preoperative PLR level was an independent factor for PFS. Therefore, we can detect high-risk patients from all gliomas patients by test PLR before treatment to receive adequate adjuvant chemotherapy. Wang et al^[6]^ and Han et al^[28]^ also got similar results like us, while Sun et al^[29]^ found the value of PLR was less effective than NLR when acting as an independent prognostic factor in prostate cancer. Our results demonstrated that low preoperative NLR suggested a poor OS and PFS, which was consistent with some studies.^[30–32]^ Furthermore, our study reconfirmed that SII, NLR, and PLR were associated with prognosis in patients with glioma.

Our study also has a number of limitations. Firstly, we included a limited number of patients, only 67 patients, including WHO I–IV gliomas. Secondly, adjuvant treatment methods covered chemoradiotherapy, chemotherapy, and radiotherapy, which may affect the outcomes. Third, the follow-up time was short and long-term survival cannot be assessed.

## 5 Conclusions

In conclusion, our research demonstrated that high pre-SII or high pre-NLR could be prognostic markers to identify glioma patients who had a poor prognosis. More studies should be carried out to verify the conclusions.

## Acknowledgments

This work was finished by the team at the Department of Radiation Oncology and Department of Neurosurgery, The Second Affiliated Hospital of Guangxi Medical University, and Thank you to all my colleagues.

## Author contributions

**Data curation**: Yuanfu Luo, Renzhi Deng, Qiulu Zhong, Xiangde Li, Xueyuan Chen, Sha Tao, Zhoubin Feng, Jiayi Liu, Yiyun Huang.

**Formal analysis**: Yuanfu Luo, Qiulu Zhong, Danjing Luo.

**Project administration**: Wenqi Liu, Jian Li.

**Writing – original draft**: Yuanfu Luo and Renzhi Deng.

**Data curation:** Yuanfu Luo, Renzhi Deng, Qiulu Zhong, Danjing Luo, Xiangde Li, Xueyuan Chen, Sha Tao, Zhoubin Feng, Liu Jiayi, Yiyun Huang, Jian Li.

**Formal analysis:** Qiulu Zhong, Danjing Luo.

**Project administration:** Wenqi Liu.

**Supervision:** Wenqi Liu.

**Writing – original draft:** Yuanfu Luo, Renzhi Deng.
